# The effectiveness of tamsulosin hydrochloride with terazosin combination therapy for chronic prostatitis Type-III b

**DOI:** 10.12669/pjms.38.3.4931

**Published:** 2022

**Authors:** Bin Duan, Xinxi Wang

**Affiliations:** 1 Bin Duan, Department of Urology, Affiliated Nanhua Hospital, University of South China, Hengyang 421002, Hunan Province, P.R. China; 2 Xinxi Wang Department of Urology, Affiliated Nanhua Hospital, University of South China, Hengyang 421002, Hunan Province, P.R. China

**Keywords:** Tamsulosin hydrochloride, Terazosin, Chronic prostatitis, Immune-mediated factor

## Abstract

**Objectives:**

To study the therapeutic effects of combined tamsulosin hydrochloride and terazosin treatment for patients with chronic prostatitis Type-III b.

**Methods:**

This study involved 180 patients with chronic prostatitis Type-III b treated between January 2018 and December 2020 conducted at Nanhua Hospital Affiliated to Nanhua University. Patients were randomly divided into two equal groups: one receiving oral terazosin hydrochloride tablets only (control group), and one orally receiving both tamsulosin hydrochloride sustained-release tablets and terazosin hydrochloride tablets (observation group). Outcome measurements included symptom scoring, inflammatory cytokine levels, as well as white blood cell and lecithin body counts in the prostatic fluid.

**Results:**

After 30 days of treatment, the observation group showed greater treatment effectiveness (86.67% vs. 73.33%, P<0.05). QLS, USS, PS, and NIH-CPSI symptom scores were lower in the observation group than the control group (P<0.05). No differences in adverse event distribution and incidence were noted. EPS IL-2 increased more in the observation group, while PGE-2, MIP-1α, and MIP-2 decreased more in the observation group. WBC levels decreased more in the observation group, while lecithin body levels increased more in the observation group.

**Conclusion:**

The combination of tamsulosin hydrochloride and terazosin for the treatment of patients with chronic prostatitis Type-III b has a significant effect. This approach reduced patient symptoms, lowered inflammatory biomarkers, and generally improved quality of life. This approach appears to have clinical value worthy of future investigation.

## INTRODUCTION

Chronic prostatitis (also known as Type-III clinical prostatitis), defined as a non-bacteria-related chronic inflammation of the prostate tissue, is caused by a variety of factors. Within the patient, chronic prostatitis is often accompanied by elevated prostatic secretions, inflammatory cells, prostate hyperplasias and other symptoms.^1^ Chronic prostatitis Type-III can be divided into Type-III a and III b, with the main symptoms for Type-III b being pain and irritation around the perineum, waist and back, lower abdomen, and urinary tract. In chronic disease, symptoms flare repeatedly, leading to a serious impact on patient quality of life.^2^ Alpha-blockers such as terazosin are commonly used to treat chronic prostatitis, resulting in improved maximum flow rate as well as reduced dysuria and urodynia.^3^,^4^ While they are well tolerated, using a single drug for a long course of treatment can have unclear effects on drug efficacy.^5^Tamsulosin blocks adrenoreceptors around the prostate, relaxes smooth muscle, relieves urethral spasm, and improves dysuria and urinary frequency.^6^ In this study, tamsulosin hydrochloride and terazosin were used to treat patients with chronic prostatitis Type-III b, and the clinical efficacy and the changes of immune mediators in prostatic fluid were investigated.

## METHODS

The records of patients with chronic prostatitis Type-IIIb who came to our hospital for treatment from January 2018 to December 2020 were selected, there were 180 patients, including 118 males and 62 females.

### Inclusion Criteria:

1. Patients diagnosed with chronic prostatitis Type-III b,^7^
2. Course of disease exceeding 3 months,
3. Patient was between 18 and 65 years of age,
4. Patients presenting the following clinical symptoms-expressed prostatic secretions (EPS) (Obtained by a urologist through prostate massage) containing white blood cell (WBC) counts ≤ 10/HP, negative EPS cultures, and pelvic region pain.

### Exclusion criteria:

1. Patients with drug addiction, alcoholism, drug abuse and mental illnesses,
2. Patients with cardiopulmonary diseases,
3. Patients with nervous system diseases, urinary system tumors, or urinary tract stenosis,
4. Patients treated with other drugs within the previous month
5. Patients with urinary system infections within the previous six months.

This study was approved by the medical research ethics committee of Nanhua Hospital Affiliated to Nanhua University (No.: 2021005; Date: April 18, 2021). The patients were randomly divided into observation and control groups of equal size. There was no significant difference in the basic characteristics between the two different treatment methods (P > 0.05) (Table-I).

**Table-I:** comparison of basic characteristics of patients with two different treatment methods.

Group	Sex(M/F)	Age(years)	Course of disease(m)
Control Group(n=90)	58/32	33.72±9.37	18.63±6.83
Observation Group(n=90)	60/30	32.96±10.21	19.04±7.72
χ^2^/t	0.098	0.517	0.379
P	0.754	0.606	0.705

Data were expressed as mean ± SD; t-test was used for between-groups analysis. Chi-square test (χ2) was used when data were expressed as percentages. P< 0. 05 indicated statistical significance.

Terazosin hydrochloride tablets (2.0 mg active ingredient per tablet, product batch numbers 20170926, 20181218, national drug standard ID: H20023659) were purchased from Shanghai Abbott Pharmaceutical Co Ltd. Tamsulosin hydrochloride sustained-release tablets (2.0 mg active ingredient per tablet, product batch numbers 171107, 181206, 190406, national drug standard ID: H20051461) were purchased from Kunming Jida Pharmaceutical Co Ltd.

### Treatment regimens

Control group patients were administered oral terazosin hydrochloride tablets, one tablet each time, one time per day. As such, the observation group was given oral tamsulosin hydrochloride sustained release tablets on the basis of the control group, one tablet each time, one time per day. Overall treatment lasted 30 days for patients in both groups.

### Observational indicators:

### Therapeutic effect

Treatment was deemed to be ineffective if: no significant change in WBC, Prostatitis symptom index, or symptoms in patients with EPS. Treatment was deemed effective if: microscopic examination of patients with prostatic fluid WBC than before treatment more than 30% of the reduction in patients with prostate diagnosis to improve the quality of patients with symptoms eased. Treatment was deemed remarkably effective if: prostatic fluid WBC count was under 30/HP, digital examination found the prostate to have become soft, and patient discomfort significantly improved. Patients were deemed to be cured if: no tenderness or discomfort was noted, and digital examination revealed that the prostate had become soft. The total effective rate of treatment = 1- (ineffective number)/total number. All operations were performed by a urologist.

### Chronic prostatitis symptom index (NIH-CPSI) score:^8^

The NIH-CPSI score consists of the quality-of-life score (QLS, a 0–12-point scale), the urinary symptoms score (USS, 0–10-point scale), pain score (PS, 0–21-point scale), for a maximum of 43 points. Patient score is directly proportional to symptom severity and inversely proportional to quality of life.

### Detection of Inflammatory Factors in EPS

Macrophage inflammatory protein-2 (MIP-2), macrophage inflammatory protein-1β (MIP-1β), interleukin-2 (IL-2), and prostaglandin 2 (PGE-2) levels were detected by radioimmunoassay (RIA) and enzyme-linked immunosorbent assay (ELISA) before and after treatment according to manufacturer’s instructions. To assess WBC counts, 20 μL of 6 U/mL heparin solution was mixed with 100 μL peripheral blood and add 10 μL filtered ink. The solution was incubated at 37°C for 4 hours, after which blood smears were prepared, dried, and stained with Wright’s stain. 100 mature neutrophils and immature neutrophils were counted under microscope, and 20 monocytes were counted too. ***-*** Prostatic fluid was collected from patients and observed under microscopy for visual confirmation of lecithin body presence. Drug-related adverse reactions, including hypotension, nausea, headache, dizziness and others, were monitored.

### Data Analysis

All data were analyzed using SPSS 18.0 software. Data were expressed as Mean ± SD, with t-tests used to examine differences between groups. Chi-square tests (χ2) were used when data were expressed as percentages. P> 0. 05 indicated statistical significance.

## RESULTS

After 30 days of treatment, the total effectiveness for the observation group was 86.67%, while the effectiveness in the control group was 73.33% (Table-II, P < 0.05). Prior to treatment, QLS, USS, PS, and NIH-CPSI symptom scores were similar among the control and observation groups. After treatment, while QLS, USS, PS, and NIH-CPSI symptom scores decreased in both groups, the decrease was greater in the observation group (Table-III, P<0.05).

**Table-II:** Therapeutic effects of the two treatment strategies.

Group	n	Ineffective	Effective	Remarkable effect	Cure	Effective rate%
Control Group	90	24	44	16	6	73.33
Observation Group	90	12	50	20	8	86.67
χ^2^						6.065
P						0.014

Data were expressed as mean ± SD; t-test was used for between-groups analysis. Chi-square test (χ2) was used when data were expressed as percentages. P< 0. 05 indicated statistical significance.

**Table-III:** NIH-CPSI Symptom Scores Before and After Treatment.

Group	n	Time	QLS	USS	PS	NIH-CPSI
Control Group	90	Before treatment	5.49±1.53	7.49±1.10	17.18±1.84	30.16±1.73
		After treatment	4.13±1.21a	5.98±1.09a	15.31±1.67a	25.42±1.47a
Observation Group	90	Before treatment	5.50±1.49	7.53±1.21	17.21±1.38	30.24±1.32
		After treatment	2.79±1.09ab	4.38±1.13ab	12.35±1.42ab	19.52±1.07ab

Data were expressed as mean ± SD; a: P < 0.05 compared against before treatment, b: P < 0.05 compared against control group; P< 0.05 indicated statistical significance.

***-*** Prior to treatment, both groups presented similar EPS levels of IL-2 and PGE-2. After treatment, EPS IL-2 levels increased in both groups, while PGE-2, MIP-1α, and MIP-2 levels decreased in both groups. The IL-2 increase, as well as the PGE-2 and MIP-1α decreases, were greater in the observation group than the control group (Table-IV, P < 0.05).

**Table-IV:** EPS IL-2 and PGE-2 levels before and after treatment.

Group	n	Time	MIP-1α (pg/ml)	MIP-2 (ng/ml)	IL-2 (ng/ml)	PGE-2(ng/ml)
Control Group	90	Before treatment	39.21±5.28	96.47±5.93	1.89±0.58	870.37±38.62
		After treatment	33.25±6.34a	85.28±6.03a	2.29±0.63a	561.28±28.93a
Observation Group	90	Before treatment	39.19±4.82	95.97±4.29	1.92±0.62	880.73±40.72
		After treatment	26.89±5.28ab	63.74±6.29ab	2.84±0.66ab	330.31±30.62ab

Data were expressed as mean ± SD; a: P < 0.05 compared against before treatment, b: P < 0.05 compared against control group; P< 0.05 indicated statistical significance.

***-*** WBC counts and lecithin body levels were similar between the two groups before treatment. After treatment, WBC levels decreased in both groups, while lecithin body counts increased in both groups. However, the magnitude of these changes was greater in the observation group (Table-V, P > 0. 05).

**Table-V:** WBC and lecithin corpuscle counts before and after treatment.

Group	n	Time	Lecithin body/HP	WBC/HP
Control Group	90	Before treatment	8.72±2.04	22.08±2.06
		After treatment	20.74±4.21 a	12.89±1.93 a
Observation Group	90	Before treatment	8.59±1.93	21.94±1.73
		After treatment	26.83±3.94 ab	6.14±1.38 ab

Data were expressed as mean ± SD; a: P < 0.05 compared against before treatment, b: P < 0.05 compared against control group; P< 0.05 indicated statistical significance.

### Adverse reactions

No differences in adverse reactions were noted between patients in the two groups during and after treatment (Table-VI).

**Table-VI:** Patient Adverse Reactions.

Group	n	Hypotension	Dizziness and headache	Nausea	Incidence Rate
Control Group	90	3	2	3	8.89
Observation Group	90	4	2	2	8.89
χ^2^					0
P					1

Data were expressed as mean ± SD; t-test was used for between-groups analysis. Chi-square test (χ2) was used when data were expressed as percentages. P< 0. 05 indicated statistical significance.

## DISCUSSION

Terazosin, as an alpha receptor blocker capable of inhibiting glandular hyperplasia and sphincter spasm, is often used in the treatment of chronic prostatitis.^9^ Alternatively, tamsulosin hydrochloride, as a selective adrenoceptor blocker, can also relieve urethral resistance and urethral spasm, improving odynuria symptoms and frequency of micturition.^10^ This study indicated that a combination of tamsulosin hydrochloride and terazosin was more effective than terazosin treatment alone. Combination therapy also resulted in lower QLS, USS, PS and NIH-CPSI scores, indicating reduced symptoms and pain. These results indicate that this novel treatment strategy could bring therapeutic benefits in terms of both treatment efficacy and patient quality of life.

The etiology of chronic prostatitis Type-III b is complicated. The condition’s main symptoms include pelvic floor striated muscle spasms, causing large numbers of microstones, inflammation, and prostatic duct dilation. The bladder neck and posterior urinary tract contain large numbers of adrenergic receptors, resulting in spasm upon stimulation. This phenomenon may occur with lower urinary tract obstruction and impacts urodynamics.^11^

Previous studies have shown that inflammatory cytokines play an important role in chronic prostatitis pathogenesis.^12^ IL-2 is a regulatory growth factor that contributes to increased PGE-2 levels upon prostatitis, leading to microvasculature dilation, increased vascular permeability, and tissue edema.^13^ MIP-2 and MIP-1α are chemotactic agents that recruit macrophages, lymphocytes and neutrophils to inflammatory sites.^14^ Previous studies have shown that patients with chronic prostatitis have higher concentrations of MIP-2 and MIP-1α in their prostatic fluid, and that the levels of these cytokines are positively correlated with the severity of chronic prostatitis.^15^ This study noted that combination therapy with tamsulosin hydrochloride and terazosin decreased IL-2, PGE-2, MIP-1α, and MIP-2 levels. This indicates that this therapeutic approach inhibited patient inflammatory responses.

The presence of WBCs in the peripheral blood, as well as lecithin corpuscles in the prostatic fluid, are signs of chronic prostatitis. Moreover, the abundance of these elements is also linked to the severity of chronic prostatitis.^16^ This study showed that the combination of tamsulosin hydrochloride and terazosin can effectively reduce the number of WBC in patients’ plasma and increase the number of lecithin bodies in patients’ EPS, which fully shows that the combination therapy can significantly improve the symptoms of patients.

However, due to the complexity of chronic prostatitis pathogenesis, this study has not fully elucidated the mechanisms by which tamsulosin hydrochloride sustained-release tablets combined with terazole acts on chronic prostatitis. The underlying genetic and molecular mechanisms need to be investigated in the future.

## CONCLUSION

The application of tamsulosin hydrochloride and terazosin in the treatment of chronic prostatitis Type-III b patients has significant effect, can significantly improve the NIH-CPSI score of patients, and can reduce the MIP-2, MIP-1 in EPS α and pge-2 level, increase the number of WBS in plasma, reduce the number of lecithin bodies in EPS, and the adverse reaction rate was not significantly increased, which has a certain clinical value.

### Authors’ contributions:

**BD** conceived and designed the study. **BD and XW** collected the data and performed the analysis. **BD** was involved in the writing of the manuscript and is responsible for the integrity of the study. **XW** edited the manuscript and made significant contribution to the study at different stages. All authors have read and approved the final manuscript.
